# Family transmission of crafts and folk art: a mixed-methods study on family relationships and cohesion among artisans in a UNESCO Creative City

**DOI:** 10.3389/fsoc.2026.1800473

**Published:** 2026-06-17

**Authors:** Sandra Igreja, Constança Paúl, Soraia Teles

**Affiliations:** 1 RISE-Health, Department of Behavioral Sciences, School of Medicine and Biomedical Sciences, University of Porto (ICBAS-UP), Porto, Portugal; 2 RISE-Health, Clinical Neurosciences and Mental Health Department, Faculty of Medicine, University of Porto, Porto, Portugal

**Keywords:** art, crafts, culture, family relations, generativity, intergenerational relations, social determinants of health, sustainable growth

## Abstract

Family encouragement and family-transmitted businesses play a crucial role in sustaining crafts and folk art across generations, serving as a key driver in safeguarding this form of intangible cultural heritage. Research on family businesses has mostly focused on business aspects, with far less attention to the family relationships and dynamics associated with them. This mixed-methods study sought to investigate how family transmission of crafts and folk art is associated with family relationships and cohesion. The research was conducted in a UNESCO Creative City of crafts and folk art in Portugal. Seventy professional artisans who reported family transmission of their practice participated in walking interviews in their ateliers and completed the Family APGAR scale, as well as a sociodemographic, professional, and family transmission questionnaire. Participants were aged 22 to 88 years (*Md* = 63), and most were male (64.3%, *n* = 45). All artisans were active in one of eight craft sectors. The majority were married /common-law (82.8%), had children (90%) and grandchildren (54.3%). Family APGAR scores ranged from 7 to 10 points, classifying families as highly functional. Significant positive correlations were found between family functioning and age (*p* = 0.041). Thematic content analysis revealed artisans’ perceptions of the positive and negative impacts of family transmission of crafts, with 11 main areas emerging in their discourse related to family relationships and cohesion. Positive implications highlighted intergenerational solidarity dynamics associated with legacy transmission and cultural heritage preservation. Overall, the generative sense of parents toward subsequent generations was evident, contributing to family cohesion and the well-being of those involved. This study suggests that family-transmitted crafts and folk art sustain cultural heritage while reinforcing family relationships and cohesion, indicating that policies supporting artisanal practices should consider the family context as relevant to their effectiveness.

## 1 Introduction

In family-based craft activities, family encouragement stands out as one of the main motivational factors. Many of these small productive units reflect a long-term orientation, sustained by dimensions of futurity, continuity, and perseverance, closely linked to the preservation of tradition (Lumpkin and Brigham, 2011; Deb et al., 2022). In recent decades, the concept of cultural heritage has expanded to encompass living expressions inherited from previous generations, such as traditional craftsmanship, whose value lies primarily in the skills and knowledge transmitted across generations, as underlined by the UNESCO Convention for the Safeguarding of the Intangible Cultural Heritage (UNESCO, 2025). UNESCO highlights that culture is an essential resource for fostering more harmonious and inclusive societies, guided by pluralism and diversity, serving as a channel for dialog and well-being (UNESCO, 2023). Embedded in individual culture, legacy has the potential to contribute personally to the future. Leaving a legacy is deeply connected to each person’s life and to how they wish to be remembered, serving as a means of transmitting a lasting image of what they represent (Hunter and Rowles, 2005).

Studies suggest that various factors contribute to the meaning of work, among which the family stands out, as it can both shape economic perceptions and provide emotional support, recognition, and appreciation, such that the meaning of work and family may influence each other reciprocally (Rosso et al., 2010). In this regard, previous studies that examined the dynamics of work and care within multigenerational families highlighted the different ways in which these families assign meaning to giving and receiving, evidencing a culture of reciprocity that accompanies a dynamic of intergenerational and supportive family transfers (Brannen, 2006). The literature has shown that older individuals find satisfaction in creating meaning and transmitting legacy; in this process, it becomes essential for the family to acknowledge and create space for this contribution to be valued in a meaningful way (Sousa et al., 2012; Serrat et al., 2021). Generativity constitutes a central component of adult development, reflecting a conscious concern for future generations. Studies have demonstrated its positive correlation with life satisfaction (McAdams and de St Aubin, 1992; McAdams et al., 1993) and have recognized it as a determinant of happiness (Shahen et al., 2019).

In the social domain, intergenerational support plays an essential role in encouraging the participation of older adults in society (Serrat et al., 2021; Wangliu, 2023). The literature shows that intergenerational engagement can benefit the well-being of the aging population, highlighting potential positive effects, such as the promotion of generativity and the improvement of attitudes and perceptions across age groups (Krzeczkowska et al., 2021). Intergenerational relationships in the twenty-first century reflect the structural evolution of families themselves, “Children learn to see older people through the eyes of their parents, teachers, peers, and the media they consume passively, and they learn to age themselves based on these surrounding images, which become ingrained and translate into behaviors and experiences in the present and future” (Paúl, 2017, p. 282). Studies that specifically analyzed intergenerational support indicate that it influences older adults’ social participation, increases life satisfaction, and promotes greater social engagement (Wangliu, 2023). Older adults, by preserving and transmitting artistic traditions, express pride and respect, improve their well-being, and ensure the continuity of art and craft practices (Tzanidaki and Reynolds, 2011). The analysis of family dynamics in crafts and folk art refers to relationships in which the transmission of knowledge, practice, and recognition circulates across generations (Igreja et al., 2025a). Previous studies with multigenerational families indicate that exchanges between generations are based on patterns of reciprocity and mutual support that express a culture of continuity and family mutuality, in which members share resources, responsibilities, and care over time (Brannen, 2006). Intergenerational solidarity has been discussed in the literature on adult families, including a theoretical model describing six dimensions of intergenerational family cohesion, association, affection, consensus, resource sharing, the strength of familism norms, and the opportunity structure for interaction; highlighting the interrelations between normative integration, affection, and association as central to understanding family cohesion (Bengtson and Roberts, 1991). Subsequent studies on intergenerational solidarity emphasize the importance of considering implicit forms of cohesion when assessing the strength and structure of family ties, noting that investigations focused exclusively on functional exchange tend to underestimate the intensity of these bonds (Silverstein and Bengtson, 1997). These dimensions allow for analysis of how family practices, such as the transmission of crafts, may be associated with cohesion across generations, mediated by affection, norms, resource exchanges, and interaction opportunities. In this context, Social Exchange Theory (Homans, 1958) serves as a complementary perspective, understanding social behavior as a process of reciprocity in which material and symbolic resources are exchanged, reinforcing group bonds and cohesion. Individual motivations are strongly shaped by the type and needs of the family, reflecting not only emotional dimensions but also the pursuit of security and identity (Barreto et al., 2014). Work-family enrichment refers to how experiences in one domain can enhance quality of life in the other, reflecting both instrumental and affective pathways (Greenhaus and Powell, 2006). The literature identifies family cohesion as one of the essential factors for the survival of family businesses (Fernández-Roca et al., 2014). Additionally, research indicates that strategies that foster family motivation can increase worker performance and engagement (Menges et al., 2016). Family relationships constitute one of the ways in which social solidarity is manifested in contemporary social arrangements (Crow, 2002). Concurrently, other studies have contributed to the literature on artisanal work, suggesting that this practice is rooted in affective relationships and organizational dynamics marked by family ties and successive generations, in which knowledge and practices are shared and transmitted over time (Bell and Vachhani, 2019). In the context of family-run artisanal enterprises, family members act as guardians of tradition, reinterpreting the past and combining cultural preservation with innovation and transgenerational entrepreneurship (Rondi et al., 2024).

Barcelos (Portugal) is a region distinguished by its extensive artisanal activity across various sectors, with a natural predominance in pottery and clay figurines, and has been designated by UNESCO as a Creative City of crafts and folk art. Crafts and folk art create a strong connection between local residents and the artisan community, contributing to the development of a sustainable creative sector. The city seeks to support the vitality of the sector and encourage a new generation of artisans (UNESCO, 2017; Município de Barcelos, 2023). This context provides an interesting setting to explore intergenerational dynamics, family transmission of craft skills, and the preservation of intangible cultural heritage.

The engagement of the next generation is one of the main contributors to the success and continuity of family businesses, and family relationships are an important factor in shaping this engagement (Garcia et al., 2019). In this regard, the literature argues that it is important to better understand how family business behavior is influenced by the social structures and relationships that emerge from the family’s involvement in the business (Zellweger et al., 2018).

Despite existing research on art and crafts, little is known about how the family transmission of these practices is associated with family relationships and cohesion. The literature dedicated to the relationship between family businesses and art and crafts is also scarce (Deb et al., 2022). Research in the field of family businesses has largely focused on the business dimension, devoting much less attention to the family relationships and dynamics associated with these enterprises, including aspects such as family functioning, well-being, communication, and family structure (Morris and Kellermanns, 2013). Moreover, gaps remain in understanding the involvement of young and adult populations in artistic activities (Fioranelli et al., 2023), despite the recognized potential of intergenerational participation to promote well-being, quality of life, and active and healthy aging (World Health Organization, 2020, 2021; United Nations, 2023; United Nations Department of Economic and Social Affairs, 2023; United Nations Department of Economic and Social Affairs, 2025; United Nations Foundation, 2025). In the case of older adults, previous studies have highlighted the lack of research exploring how artistic engagement connects with other life domains, such as family, from a life course and linked-lives perspective (Chacur et al., 2022). Another point concerns the influence of parents on the next generation’s motivation to engage in the family business, a dimension that has been little explored (Garcia et al., 2019).

A previous study with older artisans from the same Barcelos (Portugal) sample highlighted the need for more in-depth analyses of family dynamics, indicating that such an approach could allow a better understanding of the transmission of craft and folk art legacy and its impact on family cohesion (Igreja et al., 2025b). In light of these gaps, the present study aims to analyze how the family transmission of artisanal practice is associated with family relationships and cohesion, based on a sample of artisans from Barcelos (Portugal), a UNESCO Creative City of crafts and folk art (UNESCO, 2017).

## 2 Materials and methods

### 2.1 Study design

A mixed-methods, cross-sectional study was conducted. Primary data were collected through a protocol including a family functioning scale. In-depth interviews were carried out in the ateliers of professional artisans using the walking interview. This approach allows for methodological triangulation, understood as the combination of qualitative and quantitative methods to examine the same phenomenon, which constitutes a strategy that enhances the validity of the results and contributes to theoretical and scientific advancement (Morse, 1991; Hussein, 2009). Qualitative data were collected and treated inductively, enabling categories to emerge from the data. Quantitative data were analyzed using descriptive and exploratory inferential statistical analyses.

This study is part of a larger project aimed at exploring the contribution of artistic activity and the legacy of crafts and folk art to the quality of life, health status, and family cohesion of professional artisans working in Barcelos (Portugal).

### 2.2 Participants and recruitment

Data were collected from professional artisans active in ateliers located in Barcelos (Portugal), using a non-probabilistic sampling approach. Eligible participants were artisans aged 18 years or older, working in any of the following sectors: Imagery, Pottery, Embroidery, Weaving, Iron and Derivatives, Wood, Basketry and Wicker, and Contemporary Crafts. Only active professional artisans who reported family transmission of the practice, either by having inherited the activity or by having transmitted it to family members, regardless of this resulting in performing the activity professionally, were included. Potential participants were identified through public platforms, notably the official Barcelos Municipality website, where a comprehensive mapping of artisans and their ateliers is presented, complemented by artisans’ websites with an online presence. Initial contact was made by telephone, and artisans received full information about the study. All artisans meeting the eligibility criteria were invited to participate, and the data collection date was scheduled. Data collection took place in the artisans’ ateliers during the first quarter of 2024 and continued until saturation was reached that is, when no new themes emerged and the information became redundant in the in-depth interviews.

#### 2.2.1 Ethical approval and data protection

This study received a favorable opinion by the Ethics Committee of the Centro Hospitalar Universitário de Santo António, E. P. E. (CHUdSA) and the School of Medicine and Biomedical Sciences, University of Porto (ICBAS-UP) [CHUdSA/ICBAS Ethics Committee] [reference 2024/CE/P02(P418/2023/CETI)]. It was approved by the Data Protection Unit of the University of Porto (reference R-6/2025) and conducted in accordance with local legislation and institutional requirements.

All participants were provided with detailed information about the study and informed consent was obtained in writing from all participants prior to data collection.

### 2.3 Instruments

Participants completed a specially designed questionnaire to collect sociodemographic information, professional activity data, family characteristics, and a family functioning scale, the Family APGAR. The questionnaire was administered by an interviewer and complemented by in-depth interviews conducted in the participants’ natural settings. Professional artisans were asked to answer questions related to their sociodemographic characteristics, as well as to provide information about their professional activity, including the artistic sector, age at the start of the activity, location of the atelier, weekly hours dedicated to the activity, hours spent on non-professional activities, and the main benefit of the activity (economic, health, quality of life, well-being, social status). Family profile data and family members involved in the craft activity included questions such as household size; whether the artisan has children, and how many; grandchildren and great-grandchildren, and their ages; household composition; whether family members work in crafts; whether they are older and/or younger; whether the activity is regularly carried out with other generations; kinship relationships; and the number of people involved in craft activity within the family. Questions related to family transmission included whether the activity had been transmitted from previous generations and whether the artisan is currently transmitting the craft activity to younger generations.

To assess individual perceptions of family functioning, the Family APGAR scale (Smilkstein, 1978), adapted to Portugal was used (Imperatori, 1985; Agostinho and Rebelo, 1988). The scale consists of five questions, each corresponding to main family functions: 1. adaptation, referring to the use of resources, both within and outside the family, to solve problems, particularly when the family’s balance is threatened during a crisis; 2. partnership, referring to the sharing of decision-making and responsibilities among family members; 3. growth, referring to the physical and emotional maturity and self-realization achieved by family members through mutual support and guidance; 4. affection, being the expression of care or love among family members; and 5. resolve, referring to the commitment to dedicate time to other family members for physical and emotional care, typically involving decisions about sharing property and space (Smilkstein, 1978; Imperatori, 1985). Each question can be answered as “Almost always” (scoring two points); “Some of the time” (one point); and “Hardly ever” (zero points). The total score is obtained by summing the points of each question and a global score of seven to 10 points suggests a highly functional family, while four to six points suggests a family with moderate dysfunction, and zero to three points a family with severe dysfunction (Smilkstein, 1978; Imperatori, 1985). The Family APGAR shows good psychometric properties, being a reliable, validated, and useful instrument for assessing an individual’s satisfaction with family functionality, as demonstrated with different population groups (Smilkstein et al., 1982). In the three-response-option version, a Cronbach’s alpha of 0.80 was observed, and the test–retest reliability, assessed over a two-week interval, resulted in a coefficient of 0.83 (Smilkstein et al., 1982).

The Go-along Walking Interview approach was adopted, given its usefulness in obtaining contextualized information. It offers a unique way for the researcher to observe participants’ immediate environments and to explore their perceptions of how they experience their local worlds (Carpiano, 2009). A semi-structured format with open-ended questions was used, conducted in European Portuguese, based on an interview guide previously developed from the literature (Bergeron et al., 2014; Pandey and Pandey, 2015; King and Woodroffe, 2017; Sá et al., 2021). The guide included both main and secondary questions, posed according to the participants’ spontaneous discourse, thus allowing the exploration of relevant emerging themes during the interviews (see Supplementary material 1).

The interviews were audio recorded (1 to 2 h each), fully transcribed, and complemented with observations and field notes. The complete recordings were listened to at least twice to become familiar with the content and with the participants’ discursive particularities, while annotating elements considered useful for writing and analyzing the field notes, which formed part of the analytical process.

### 2.4 Data analysis

#### 2.4.1 Statistical analysis

Numerical data were analyzed using SPSS software, version 29. Descriptive statistics were calculated according to the nature and distribution of the data. To explore associations between Family APGAR scores and sociodemographic, professional, and family variables, non-parametric tests were used. Differences between groups were analyzed using the Mann–Whitney *U* test for non-parametric continuous or ordinal variables. Associations between continuous or ordinal variables were examined using Spearman’s rank correlation coefficient, according to the nature of the data. All statistical tests were two-tailed, and the level of significance was set at 0.05.

#### 2.4.2 Content analysis

A thematic content analysis of text data was conducted, following a horizontal scheme (Bardin, 2011). NVivo software (Release 1.7.2) was used to code the sources. To monitor data saturation, preliminary observations were carried out simultaneously with data collection. Saturation was considered to have been reached when no new themes emerged, and redundancy was observed in participants’ responses. One interview conducted shortly after the saturation point (previously scheduled during quantitative data collection) was also considered. After this interview, no further recruitment efforts were made, and data collection ceased. At that point, the percentage of participants was mapped in comparison with the reality of the territory. Data were grouped into categories and subcategories based on a bottom-up, data-driven, inductive approach.

To enhance the validity of the study, two researchers on the team (A and B) conducted the data analysis and coding. To ensure that text units were preserved for both coders, the data were first segmented by researcher A. The coding tree was initially developed by researcher A and subsequently tested by researcher B. Researcher B did not participate in participant recruitment, data collection, or interview transcription. Inter-coder agreement was assessed qualitatively. The initial agreement was examined, both coders compared results, uncertainties in coding were discussed, and discrepancies were resolved through joint analysis. Interactions between researchers A and B then followed to refine the coding tree, as well as the definitions of each category and subcategory. Based on the consensus reached, the final version of the coding tree was established.

The combination of independent coding with multiple data collection methods, including walking interviews, field notes, and a comprehensive questionnaire, constituted a triangulation process, contributing to a more robust representation of the phenomena under study (Bengtsson, 2016).

Various procedures were carried out during the thematic content analysis, including word clouds, hierarchy charts and cross-coding analyses between themes and participant attributes, aiming to identify patterns. Absolute and relative frequencies of coded references in the categories were cross-tabulated with participant attributes (age, gender, education, retired/non-retired, marital status, number of children, craft sector, age of entry into the craft, and weekly hours dedicated) to provide a more comprehensive view of their perceptions. The analysis primarily focused on participants’ narratives, which constitute the central basis for interpretation, with cross-reference tables used solely to support the reading of the narratives. The narratives in each subcategory were examined in detail and combined with participant attributes to help clarify or deepen observed trends, serving to contextualize and expand understanding of the implications of craft practice in family life. The inclusion of certain attributes was supported by the Family APGAR scale results, which indicated statistically significant differences according to age and retirement status.

Proportions of references by attribute were calculated based on the total number of references for each participant group (e.g., retired/non-retired). For instance, the proportion of references to the subcategory “The family environment linked to craft as an enhancer of family relationships and cohesion” (N1.3) in each group was determined by dividing that number by the total number of references coded in the respective group and multiplying the result by 100. This procedure allowed the analytical categories to participants’ contexts and identified patterns in their perceptions of family relationships and cohesion.

The results are presented as: (a) absolute frequencies of references coded per node/(sub)category, and (b) absolute frequencies of artisans coded per node/(sub)category. The hierarchy chart included in the study visually represents the number of cases coded per category/coding node. Text excerpts are included to illustrate participants’ perceptions related to each subcategory.

The Consolidated Criteria for Reporting Qualitative Research (COREQ) (Tong et al., 2007) were followed for this manuscript (see Supplementary material 2).

## 3 Results

### 3.1 Statistical analysis (quantitative data)

#### 3.1.1 Sociodemographic and professional characteristics

Table 1 presents the participants’ sociodemographic characteristics, and Table 2 shows variables related to professional characteristics. Seventy professional artisans (*n* = 70) participated in the study, representing 34.83% of the total number of artisans in the territory (*n* = 201), according to information from the official municipal website (Município de Barcelos, 2023).

**Table 1:** Sociodemographic characteristics of participants.

Variables	*N*	Descriptive statistics
**Age** (years), M (SD)	70	61.64 (13.33)
**Gender**, Male, *n* (%)	70	45 (64.3)
**Marital status**, *n* (%)	70	
Married / Common-law		58 (82.8)
Widow(er)		5 (7.1)
Single		5 (7.1)
Divorced		2 (2.9)
**Years education**, M (SD)	70	7.49 (3.90)
**Professional training**, Yes, *n* (%)	70	19 (27.1)
**Retired**, Yes, *n* (%)	70	32 (45.7)
**Crafts as main professional activity**, yes, *n* (%)	70	62 (88.6)
**Main source of income**, *n* (%)	70	
Crafts		69 (98.6)
Pension/Retirement		31 (44.3)
Other income		9 (12.9)
**Monthly income**, based on the National Minimum Wage (NMW), *n* (%)	70	
Monthly income ≤ NMW		39 (55.7)
Monthly income > NMW		31 (44.3)

**Table 2:** Professional characteristics of participants.

Variables	*N*	Descriptive statistics
**Craft sector**, *n* (%)	70	
Imagery		35 (50.0)
Pottery		11 (15.7)
Iron and derivatives		6 (8.6)
Wood		5 (7.1)
Embroidery		4 (5.7)
Contemporary crafts		4 (5.7)
Basketry and wicker		3 (4.3)
Weaving		2 (2.9)
**Age of entry into the craft sector**, *M* (*SD*)	70	17.17 (13.75)
**Years of residence in the parish**, *M* (*SD*)	70	51.13 (21.20)
**Atelier location at the residence**, *n* (%)	70	56 (80.0)
**Weekly hours dedicated to craft activity**, *M* (*SD*)	70	50.04 (15.62)
**Weekly hours dedicated to non-professional activities**, *M* (*SD*)	70	9.40 (8.64)
**Perceived main benefits of involvement in the craft activity**, *n* (%)	70	
Well-being		55 (78.6)
Economic		25 (35.7)
Health		16 (22.9)
Quality of life		16 (22.9)
Social status		9 (12.9)

The participants’ ages ranged from 22 to 88 years, with a median of 63 years. The majority were male (64.3%, *n* = 45) and married/common-law (82.8%, *n* = 58). Years of education ranged from 0 to 19, with 32.9% (*n* = 23) of participants having completed up to 4 years of schooling and 28.6% (*n* = 20) between 7 and 9 years. A total of 27.1% (*n* = 19) reported having professional training. Thirty-two artisans were formally retired (45.7%), and 88.6% (*n* = 62) considered craftwork their main occupation. For nearly all participants (98.6%, *n* = 69), craftwork constituted the primary source of income, while 44.3% (*n* = 31) also reported receiving a pension/ retirement. The majority of artisans (55.7%, *n* = 39) had a monthly income equal to or below the national minimum wage. Participants had a median of 55 years of residence in their parish, with most living in owned houses (94.3%, *n* = 66), and 80% (*n* = 56) had their ateliers at their place of residence. The artisans were distributed across eight sectors of crafts and folk art. Half of the participants (50%, *n* = 35) were engaged in the Imagery sector, while the other sectors account for the remaining participants. In the embroidery sector, participants highlighted Crivo Embroidery of São Miguel da Carreira. This certified production presents unique characteristics distinguishing it from other local embroideries and preserves a centuries-old tradition specific to a particular area of the territory (Município de Barcelos, 2023). Involvement in craft activities began by age 10 for 41.4% of participants, and between ages 11 and 20 for 38.6%. Weekly, 82.9% (*n* = 58) of artisans dedicated more than 40 h to craftwork, with a range from 18 to 96 h and a mean of 50.04 h (SD = 15.62). Time spent on non-professional activities averaged only 9.40 h per week (SD = 8.64). Among the main perceived benefits of working in the crafts and folk art sector, well-being was by far the most frequently cited (78.6%, *n* = 55), whereas social status was the least reported benefit (12.9%, *n* = 9).

#### 3.1.2 Family characteristics and functioning

The average number of people per household was 2.80 (SD = 1.05), ranging from 1 to 5. The most common household sizes were two (50%, *n* = 35) and four members (30%, *n* = 21). Single-person and five-member households were less frequent (4.3%, *n* = 3 each). Regarding household composition, most artisans live with their spouse or partner (88.6%; *n* = 62). Almost half (45.7%; *n* = 32) also reported living with their children.

Among the participants, 90% (*n* = 63) reported having children, with a median of two (range 1–7). Most had two children (50.8%; *n* = 32), followed by three (22.2%; *n* = 14) and one child (11.1%; *n* = 7).

More than half of the artisans (54.3%; *n* = 38) reported having grandchildren, with a median of four (range 1–19). The most frequent numbers were two and four grandchildren (18.4%; *n* = 7 each). Only a small proportion (5.7%; *n* = 4) reported having great-grandchildren, with a median of five.

The age of sons and daughters ranged from 0 to 70 years, with most between 21 and 50 years. Grandchildren were aged between 0 and 40 years, most commonly up to 20 years. Great-grandchildren were all under 10 years old. Most participants (78.6%; *n* = 55) reported having family members working in crafts. Among those with family in the activity, the median was 2 family members per participant; 47.3% (*n* = 26) reported younger family members, 29.1% (*n* = 16) indicated having both older and younger family members, and 23.6% (*n* = 13) reported only older family members.

All total scores on the Family APGAR scale (with a minimum possible score of 0 and a maximum possible score of 10) fell within the cut-off range of 7 to 10 points, classifying families as highly functional. The median score was 10, and most artisans (84.3%; *n* = 59) obtained the maximum score of 10 points. These results suggest a ceiling effect in the scale, reflected by the concentration of scores at the upper limit.

Sociodemographic, professional, and family-related variables were examined in relation to Family APGAR scores. Analysis revealed a positive correlation between age and family functioning (*n* = 70, *r_s_* = 0.245, *p* = 0.041), suggesting that older participants tended to report higher levels of family functioning. Moreover, retired participants reported higher levels of family functioning (*n* = 32; Mean Rank = 38.81) compared to non-retired participants (*n* = 38; Mean Rank = 32.71; *U* = 502.00; *Z* = −1.982; *p* = 0.047).

Table 3 presents the information collected on family characteristics and functioning (*n* = 70).

**Table 3:** Family characteristics of participants and members involved in craft and folk art activities, and perception of family functioning (Family APGAR scale).

Variables	*N*	Descriptive statistics
**Household size**, *M* (SD)	70	2.80 (1.05)
**Offspring**, yes, *n* (%)	70	63 (90)
**Number of offspring**, *M* (SD)	63	2.62 (1.34)
**Grandchildren**, yes, *n* (%)	70	38 (54.3)
**Number of grandchildren**, *M* (SD)	38	4.89 (3.71)
**Great-grandchildren**, yes, *n* (%)	70	4 (5.7)
**Number of great-grandchildren**, *M* (SD)	4	8.50 (10.14)
**Household composition**, *n* (%)	70	
Spouse or partner		62 (88.6)
Sons and daughters		32 (45.7)
Lives alone		3 (4.3)
**Family members involved in craft activities**, *M* (SD)	70	1.61 (1.06)
**Regular engagement in craft activities**, *n* (%)	70	
Intergenerational (with other generations)		30 (42.9)
Intragenerational (with the same generation)		19 (27.1)
Works alone		21 (30)
**Regular engagement in craft activities by kinship relationship**, *n* (%)	49	
Collateral or in-law relatives (siblings, spouse)		23 (46.9)
Descendant relatives		15 (30.6)
Ascendant relatives		8 (16.3)
Ascendant and collateral relatives		2 (4.1)
Engagement with non-relative		1 (2.0)
**Family APGAR scale**	70	
APGAR score, *Md* (IQR)		10 (0)
APGAR score 7–10: highly functional family, *n* (%)		70 (100)

### 3.2 Content analysis (qualitative data)

The walking interviews conducted in the ateliers generated a total of 259,302 transcribed words, supplemented by field notes. A total of 466 text units were coded, resulting in a coding tree composed of three “parent” nodes (categories) and 11 main subcategories. The first category, Positive implications of family transmission of craft practice on family relationships and cohesion (N1), includes six subcategories (N1.1; N1.2; N1.3; N1.4; N1.5; N1.6); the second, Negative implications of family transmission of craft practice on family relationships and cohesion (N2), comprises three subcategories (N2.1; N2.2; N2.3); and the third category, Implications of lack of continuity of craft practice on family relationships and cohesion (N3), contains two subcategories (N3.1; N3.2) addressing both negative and positive implications of discontinuity. Additionally, four of the “child” categories were further divided into sub-subcategories for a more detailed analysis, resulting in 10 subordinate subcategories: four within N1 and six within N3.

Table 4 presents the coding tree and definitions of the categories on the implications of family transmission for family relationships and cohesion, as well as the absolute frequencies of cases and coded references per category and subcategory. Excerpts from participants’ narratives have been included, translated from European Portuguese into English. Although minor variations may exist between the language versions, none alters the original meaning of the statements.

**Table 4:** Coding tree and category definitions, including absolute frequencies of cases and coded references by parent and child nodes/categories, and illustrative excerpts coded in each category.

Category	Description	Cases (n)	References (n)	Excerpts (examples)
N1_Positive implications of family transmission of craft practice on family relationships and cohesion	The text units in this category refer to the positive implications associated with the family transmission of craft practice, highlighting the strengthening of intergenerational relationships, the promotion of family cohesion, the sharing of knowledge, and the preservation of cultural heritage within the family.	63	342	(see child nodes)
N1.1_ Family collaboration and solidarity in craft practice	The text units in this subcategory illustrate cooperation, solidarity, and support among family members in craft practice, demonstrating their role in strengthening relationships and family cohesion: Intergenerational family collaboration and solidarity in craft practice (N1.1.1); Intragenerational family collaboration and solidarity in craft practice (N1.1.2).	43	105	N1.1.1 “My daughter has been helping me, she helps me just like my wife does, although of course she is following her own path. She, my daughter, is out there pursuing her own path, she helps me but she is following her own path in terms of her academic training” (Contemporary Crafts, 55 years old, male); N1.1.2 “Then it has an advantage because I do it in a certain way, and then she [referring to his wife] paints it according to what I made. She gives it the kind of painting that more or less brings it to life, and that’s what makes it… well, that’s how it gets done. She completes what I wanted to do” (Imagery, 76 years old, male).
N1.2_Continuity of the family legacy of craft and folk art in strengthening family relationships and cohesion	The text units in this subcategory highlight the continuity of craft practice within the family, the value attributed to inherited know-how, immersion and ongoing practice, and the desire to preserve the craft legacy, reinforcing relationships and cohesion among family members.	38	68	“I think, at least I would be happy to have someone follow in my footsteps, you know, just like my father did. He passed on what he knew, instilled it in me, and it must be a source of pride to know that someone continues it. And I think I would also feel proud if someone, even if not family, wanted to learn. There’s no problem in teaching. No, I do not want to keep any secrets to myself. Everything I can teach, I teach; there’s nothing you cannot learn” (Imagery, 46 years old, male).
N1.3_The family environment linked to craft as an enhancer of family relationships and cohesion	The text units in this subcategory refer to the family environment associated with craft practice as a rooted and enduring element, marked by involvement in the craft, which enhances relationships and cohesion among family members.	34	54	“During my youth, I took on some roles in my parents’ pottery because, well, it was normal, as you grew up and gained some maturity, they would give you certain responsibilities. Look, now you wrap this pottery, or you pack it; I went through all the processes… In terms of creativity, I’m very aware that a large part of my creativity comes from having grown up in the environment I did” (Pottery, 38 years old, male).
N1.4_Valuing, pride, and family recognition of craft work	The text units in this subcategory refer to valuing, pride, and family recognition associated with craft work, highlighting the implications of these feelings for relationships and cohesion among family members: Intergenerational valuing, pride, and family recognition of craft work (N1.4.1); Intragenerational valuing, pride, and family recognition of craft work (N1.4.2).	23	41	N1.4.1 “So, I think what defines our roosters, what we believe, my father, my wife and I, is that even though our rooster is a traditional Barcelos one, it has a very different painting. So, anyone who knows us can tell it was made by us, marking the difference, it’s our identity. I think that’s it… The children come here, they like to look, not to work, but to look and appreciate it. Sometimes they like to have our pieces at home. ‘It’s nice to have it at home!’ It’s about valuing it” (Imagery, 46 years old, male). N1.4.2 “It has meaning, because when you pass on a legacy, when you are with someone who watches and is also interested in what we do, you know, when they could just be comfortably at home. And I taught her, and she learns well. I wish that everyone I bring here had her kind of dedication, not just because she’s my partner… She really likes crafts” (Basketry and Wicker, 65 years old, male).
N1.5_Affective memory and multigenerational transmission of the craft legacy	The text units in this subcategory highlight affective memories of the craft legacy across different generations of the family, as well as the implications of these experiences for family relationships and cohesion.	17	28	“My story started with this [pointing to the hedgehogs he makes on the table at the atelier]. My mother… so she would gather us here at the table, and we had to make this and put it next to her, that’s the little spikes for the hedgehogs. We did it like this, and she would just pick them up and glue them. It started like that, but that was when we were kids. Later on, I started, I started sitting next to her to help, and over time, I kept staying. I stayed” (Imagery, 55 years old, male).
N1.6_Economic sustainability and family resilience through craft practice and the preservation of cultural heritage	The text units in this subcategory refer to the contribution of craft practice to economic sustainability and to resilience across generations, demonstrating its implications for the preservation of cultural heritage and for strengthening family relationships and cohesion.	18	21	“It means that, first, I really love the craft, the area I work in, and second, knowing that we have several ancestors who worked in this, who made their living from it, and that’s how we lived and earned our income. To continue it, and to feel that my parents are happy because we keep working and do not let the tradition die, and so that our grandparents, if they were still here and could see something, would feel that someone is carrying on what they kept going, that’s something we think about a lot. And of course, to feel some return, because if there’s no return, the bills still have to be paid” (Pottery, 59 years old, male).
N2_Negative implications of family transmission of craft practice on family relationships and cohesion	The text units in this category refer to the negative implications associated with the family transmission of craft practice, highlighting challenges that affect relationships and cohesion among family members: difficulties in continuity; low income and long working hours; and difficulties in balancing craft practice with family life.	28	42	(See child nodes)
N2.1_Difficulties in continuity despite transmission	The text units in this subcategory refer to difficulties in maintaining the continuity of craft activity among family members, reflecting concerns and uncertainties about the future of the practice and its implications for family relationships and cohesion.	11	14	“My sister worked, but only a little. Due to health issues, she had to stop” (Imagery, 74 years old, female).
N2.2_Low income and long working hours	The text units in this subcategory refer to low income and long working hours in craft activity, highlighting their negative impacts on family relationships and cohesion.	11	13	“Life is not easy. For example, I’ve probably already made thousands of pieces, but if I only lived off this, no, I could not, it’s true. I’m forced to have another job because the cost of living is very high” (Wood, 57 years old, male).
N2.3_Difficulties in balancing craft practice and family life	The text units in this subcategory refer to difficulties in balancing the time and demands of craft practice with family relationships and responsibilities, evidencing negative implications for family relationships and cohesion.	12	15	“This is an activity that keeps us away for long periods. It’s like this, I have a good relationship with my family, but it’s not easy. We have to be very flexible, because often we are here until nine or ten pm finishing a job. We’re not at home with the family. Many times we go to fairs, craft fairs around the country” (Imagery, 51 years old, male).
N3_Implications of lack of continuity of craft practice on family relationships and cohesion	The text units in this category refer to the implications of the lack of continuity of craft practice within the family context, highlighting how the absence of the practice can affect relationships and cohesion among family members, including both negative and positive impacts.	37	82	(See child nodes)
N3.1_Negative implications of lack of continuity	The text units in this subcategory refer to the negative implications of the lack of continuity of craft practice, highlighting multiple causes: Disinterest or disengagement of descendants (N3.1.1): refers to the lack of interest or aptitude of descendants in engaging in craft practice, including indifference or distancing; Low profitability and attractiveness of the activity (N3.1.2): refers to the low profitability and limited attractiveness of the activity, considering factors such as long working hours and specific characteristics of the practice; Loss of continuity of the legacy (N3.1.3): refers to concerns about the continuity of craft practice, highlighting the perception of loss of the family legacy; Conflict between respecting choices and the desire for continuity (N3.1.4): refers to the emotional conflict of parents between respecting descendants’ choices and wishing for the continuation of craft practice.	34	67	N3.1.1 “I do not have anyone, because in my family it’s like this: my children do not want to come here, and I would like this to continue in the future, with new people interested in learning, but I do not really see that happening” (Imagery, 64 years old, female). N3.1.2 “People are drawn to it because they like it, they think it’s beautiful, but then, when they see how much work it really takes, they get discouraged. This is a job that requires a lot of effort, but it does not bring in much money; that’s why my children did not want to do it… For example, I had enough work to hire one or two employees, but in the end, there is not enough money to pay them. The money just does not go far, because this does not make the kind of money people think it does” (Imagery, 71 years old). N3.1.3 “It means sadness, because I will insist until the very last day that there is someone to continue. I regret this ending and feel sad about it. I feel joy in what I do, sadness that there’s no one else, and that’s why I say I will insist until the last day. And I have hope, I have hope, I do not know, it seems impossible that this will end” (Weaving, 71 years old, male). N3.1.4 “I do not see my children working here; they come here, but unless the world takes a turn… who knows? This is an art, and an art is always good for someday. But I do not really see them doing this. As for my grandchildren, I do not know…” (Basketry and Wicker, 65 years old, male).
N3.2_Positive implications of lack of continuity	The text units in this subcategory refer to the positive implications of the lack of continuity of craft practice, highlighting: Valuing the individual vocation (N3.2.1): refers to the recognition that each descendant follows their own vocation or interest, reflecting respect for individual paths, preferences, and interests of the descendants; Sense of protection for descendants (N3.2.2): refers to interpreting the lack of continuity as something natural or protective, showing that parents perceive craft practice as a demanding activity, with low profitability or limited future prospects.	10	15	N3.2.1 “It’s like this: with the younger generation, it all depends on interest, you know, it depends on what they like. We should never, never pressure them, so to speak. The younger generation needs to have that talent; they need to have the desire to be here” (Imagery, 53 years old, male); “It’s difficult to teach this; you realize that each person is born for their own art. My eldest son and the girls all worked in ceramics here at home until they got married. After they married, each went on with their own life” (Imagery, 84 years old, male). N3.2.2 “I also have two children, but they pursued other careers; they studied. Why? Well, as I was saying, both pottery and these more artisanal activities have only begun to receive recognition in the past few years. (.) And of course, as a parent, I would never tell my children to come here” (Pottery, 58 years old).

Each excerpt includes the artisan’s attributes: craft and folk art sector, age, and gender. N = Node; n = Number (cases/references). The gray tone is applied to the main categories (“parent” nodes).

**Figure 1:**
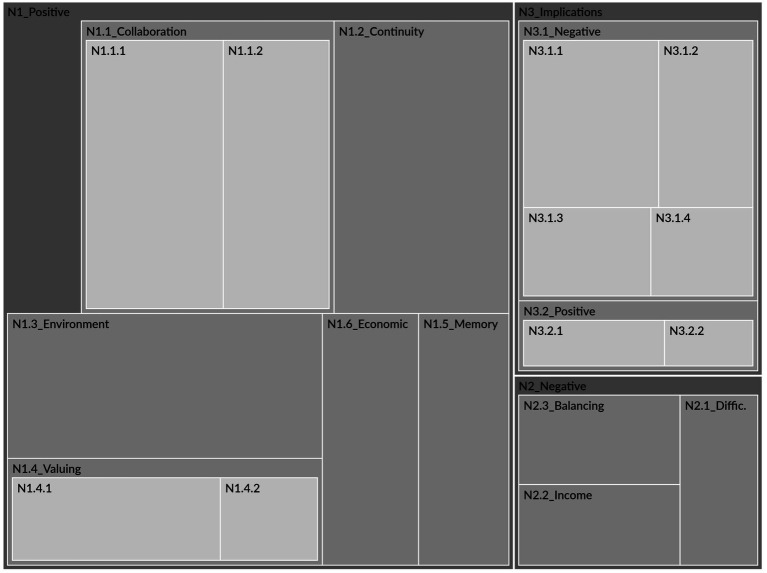
Visual representation of the number of cases/participants per category/coding node. Dark gray areas represent “parent” nodes, and mild gray areas represent “child” nodes. N1 = Positive implications of family transmission of craft practice on family relationships and cohesion; N1.1 = Family collaboration and solidarity in craft practice; N1.1.1 = Intergenerational family collaboration and solidarity in craft practice; N1.1.2 = Intragenerational family collaboration and solidarity in craft practice; N1.2 = Continuity of the family legacy of craft and folk art in strengthening family relationships and cohesion; N1.3 = The family environment linked to craft as an enhancer of family relationships and cohesion; N1.4 = Valuing, pride, and family recognition of craft work; N1.4.1 = Intergenerational valuing, pride, and family recognition of craft work; N1.4.2 = Intragenerational valuing, pride, and family recognition of craft work; N1.5 = Affective memory and multigenerational transmission of the craft legacy; N1.6 = Economic sustainability and family resilience through craft practice and the preservation of cultural heritage. N2 = Negative implications of family transmission of craft practice on family relationships and cohesion; N2.1 = Difficulties in continuity despite transmission; N2.2 = Low income and long working hours; N2.3 = Difficulties in balancing craft practice and family life. N3 = Implications of lack of continuity of craft practice on family relationships and cohesion; N3.1 = Negative implications of lack of continuity; N3.1.1 = Disinterest or disengagement of descendants; N3.1.2 = Low profitability and attractiveness of the activity; N3.1.3 = Loss of continuity of the legacy; N3.1.4 = Conflict between respecting choices and the desire for continuity; N3.2 = Positive implications of lack of continuity; N3.2.1 = Valuing the individual vocation; N3.2.2 = Sense of protection for descendants. Short keywords were added inside each node to facilitate overall interpretation.

The hierarchy chart shown in Figure 1 illustrates the distribution of categories and subcategories in terms of the number of participants/cases who mentioned each theme, providing an overview of the coding coverage. The size of each area represents the number of participants who mentioned the theme: the larger the area, the greater the number of participants addressing that theme.

Based on the hierarchy chart (Figure 1) and the coding tree (Table 4), the detailed analysis is presented in the following subsections.

#### 3.2.1 Positive and negative implications of family transmission of craft practice on family relationships and cohesion

“Positive implications of family transmission of craft practice on family relationships and cohesion” (N1) were identified by the majority of participants (90%; *n* = 63). Some subcategories stood out for emerging in the narratives of most participants: “Family collaboration and solidarity in craft practice” (N1.1: n_cases_ = 43, n_references_ = 105) and “Continuity of the family legacy of craft and folk art in strengthening family relationships and cohesion” (N1.2: n_cases_ = 38, n_references_ = 68). Nearly half of the participants referred to the subcategory “The family environment linked to craft as an enhancer of family relationships and cohesion” (N1.3: n_cases_ = 34, n_references_ = 54).

Other subcategories emerged from participants’ narratives, although mentioned by a smaller number of artisans, including: “Valuing, pride, and family recognition of craft work” (N1.4: n_cases_ = 23, n_references_ = 41); “Affective memory and multigenerational transmission of the craft legacy” (N1.5: n_cases_ = 17, n_references_ = 28); and “Economic sustainability and family resilience through craft practice and the preservation of cultural heritage” (N1.6: n_cases_ = 18, n_references_ = 21).

“Negative implications of family transmission of craft practice on family relationships and cohesion” (N2) were mentioned by 40% of participants (n = 28). Three subcategories emerged, each mentioned by a minority of participants and references, with similar values: “Difficulties in continuity despite transmission” (N2.1: n_cases_ = 11, n_references_ = 14); “Low income and long working hours” (N2.2: n_cases_ = 11, n_references_ = 13); “Difficulties in balancing craft practice and family life” (N2.3: n_cases_ = 12, n_references_ = 15).

Within the domain of positive implications (N1), the subcategory “Family collaboration and solidarity in craft practice” (N1.1) was the most frequently mentioned by the artisans, in terms of cases reflecting solidarity and support among family members in craft practice. Additionally, the data supported the creation of two child subcategories (“child” nodes), capturing both intergenerational (N1.1.1: n_cases_ = 31, n_references_ = 50) and intragenerational dimensions (N1.1.2: n_cases_ = 24, n_references_ = 55). On the one hand, the text units illustrate support among family members of different generations, such as parents and children or grandparents and grandchildren. This support manifests both in the transmission of techniques and in providing the physical conditions necessary for craft practice, as exemplified by the statement: “I work in my father’s atelier, and everything is at my disposal, and he helps us in that regard. And if it were not for that, it would have been very difficult to get started, I admit” (Imagery, 31 years old, male). In the narratives, support from children also emerged, even when they pursued other professional paths, either by helping during periods of heavier workload or as a means of shared experience, fostering family relationships and intergenerational affection, as the statement illustrates: “They all enjoy it, we get together here every Saturday or Sunday… they like coming, the youngest likes to come and paint, and the older girl enjoys making pieces” (Imagery, 72 years old, female). On the other hand, support among family members of the same generation in craft practice, such as siblings or spouses, highlights its role in strengthening family relationships and cohesion. Situations of collaboration among family members were described, both in the joint creation of pieces and in sales/fairs: “We work together, he makes his pieces, then we go paint, we paint together. He does the eyes, he does the hair, that’s his part” (spouse, Imagery, 64 years old, female); “I’m always here working, pretty much. I’m her right hand, I really am. I like working, I have fun working” (spouse, Imagery, 68 years old, male).

Regarding the subcategory “Continuity of the family legacy of craft and folk art in strengthening family relationships and cohesion” (N1.2), the narratives highlight both the appreciation of continuing traditional themes and the perception that clients still seek out these practices: “I keep making the themes, look, by chance I’m even making the ones my great-grandmother used to make, my mother carried it on. For example, my mother made this piece, and I keep making it because people ask for it” (Imagery, 55 years old, male). In addition, the inherited know-how and learned techniques are valued, along with the willingness to preserve the legacy: “It’s a characteristic feature, you see, that I learned from my mother, the little water birds… And I learned it from my mother” (Imagery, 46 years old, male). When crossing subcategory N1.2 with the attribute “number of children,” it was observed that continuity of the family legacy was more frequently mentioned by participants with four children (75%, 3 out of 4), three children (64.3%, 9 out of 14), and by those with one child (57.1%; 4 out of 7).

The subcategory “The family environment linked to craft as an enhancer of family relationships and cohesion” (N1.3) stood out in the narratives through the sense of family belonging that emerges from the craft activity. Participants reported that family involvement motivated the learning of the craft and reinforced the perception of a shared and enduring practice associated with intergenerational closeness, as illustrated by the statement referring to the grandmother: “We’d come back from school, and she would have us by her side, teaching us how to make the clay figurines. We grew up, always learning from her, as if she were our teacher of this activity. So, the path was always school, then work” (Imagery, 51 years old, male). In addition to the role of ascendants, it was also observed that initiation into craft practice occurred in some cases by a spouse. When crossing subcategory N1.3 with the attribute retired/not retired, it was observed that it was mentioned by 55.3% of non-retired participants (*n* = 21 out of 38) and by 40.6% of retired participants (*n* = 13 out of 32).

In the subcategory “Valuing, pride, and family recognition of craft work” (N1.4), two child subcategories emerged: “Intergenerational valuing, pride, and family recognition of craft work” (N1.4.1: n_cases_ = 17, n_references_ = 32) and “Intragenerational valuing, pride, and family recognition of craft work” (N1.4.2: n_cases_ = 8, n_references_ = 9). The narratives primarily revealed themes related to relationships among family members from different generations, such as parents and children or grandparents and grandchildren, emphasizing both the appreciation and recognition of the work produced, as well as the desire to possess the crafted pieces, as illustrated by the excerpt: “They could not do it, but they like it a lot. I have daughters who admire this, and everything I have, they have too, a piece of each in their house, and I like that. It makes me really happy, it’s true” (Imagery, 71 years old, female). Although less frequently, appreciation within the same generation also emerged, expressed through recognition among siblings and spouses: “I have my wife, who is also an artisan, and I can, and I’ll say it, she’s amazing at creating. She creates a piece, you put a theme in front of her, and she makes it. I might slightly improve it afterward, but she’s really good at creating. It’s true” (Imagery, 68 years old, male).

In the subcategory “Affective memory and multigenerational transmission of the craft legacy” (N1.5), the artisans expressed an affective connection linked to various moments of their interactions with family members, particularly ascendants. References emerged to artifacts used by family members in the craft practice, as well as to memories of expressions they used to say. Participants recounted shared experiences in carrying out the craft activities, keeping intergenerational memory and affection alive and highlighting the importance these experiences had in their engagement with the practice: “Now I remember, when I used to watch him sculpt, I found it interesting, what he was doing, I mean, with his hands, what he was doing. And I, his calmness while sculpting, his concentration… so there he was sculpting, and I felt that curiosity too, wanting to try it, because I remember there were things I could not do, that I did not do, but I remember smaller things mainly, and I thought, I’m not able to do that! And then I tried, and sometimes, of course, it came out more or less similar. But well, that’s how people started” (Imagery, 62 years old, male).

Regarding the subcategory “Economic sustainability and family resilience through craft practice and the preservation of cultural heritage” (N1.6), several artisans reported making a living from their activity, considering it a source of family support: “I raised my children with this. Now it gives a little more than it used to” (Pottery, 78 years old, male). When crossed with the retired/non-retired attribute, greater expression was observed among non-retired participants (34.21%, *n* = 13) compared to retired participants (15.62%, *n* = 5).

In the domain of negative implications (N2), the subcategory “Difficulties in continuity despite transmission” (N2.1) reflects concerns and uncertainties regarding the future of the practice, highlighting vulnerabilities such as health problems, a reduction in activity, and a limited number of family members currently involved in the craft: “It’s been very quiet; since my wife passed away, I have not worked here at all” (Wood, 81 years old, male). On the other hand, the subcategory “Low income and long working hours” (N2.2) highlighted situations of high dedication with low financial return: “It works for those who are at home, because we do not keep track of hours; for us, it’s from morning until midnight, one o’clock, two. Sometimes she goes to bed at 2 or 3 in the morning to make the most of it, of course. During the eight-hour workday, little or nothing gets done… It’s not very profitable, the wages are always low” (Imagery, 76 years old, male).

Finally, the subcategory “Difficulties in balancing craft practice and family life” (N2.3) highlights challenges in reconciling the demands and time required for craft practice with family relationships and responsibilities. Some children expressed concern about excessive work, noting that their parents should not work so much, and similar situations were reported between spouses: “I focused on the work, from morning till night, and I never went out; I always said I did not have time. My husband would always say, ‘You’ll go when you are old, when you cannot walk.’” (Crivo Embroidery of São Miguel da Carreira, 69 years old, female).

#### 3.2.2 Positive and negative implications of lack of continuity of craft practice on family relationships and cohesion

The “Implications of lack of continuity of craft practice on family relationships and cohesion” (N3) emerged among the majority of participants (52.85%; *n* = 37), and the coded references in this category accounted for 17.59% (*n* = 82) of the total. Almost half of the participants mentioned the subcategory “Negative implications of lack of continuity” (N3.1: n_cases_ = 34, n_references_ = 67), corresponding to 48.57% of participants and 14.37% of coded references. In turn, the subcategory “Positive implications of lack of continuity” emerged in a minority of participants and references (N3.2: n_cases_ = 10, n_references_ = 15), representing 14.28% of participants and 3.21% of coded references.

The subcategory “Negative implications of lack of continuity” (N3.1) comprises four child nodes including, disinterest or disengagement of descendants, low profitability and attractiveness of the activity, loss of continuity of the legacy, and conflict between respecting individual choices and the desire for continuity of the family practice. Among these, “Disinterest or disengagement of descendants” was the most prominent theme (N3.1.1: n_cases_ = 20, n_references_ = 30), reflecting descendants’ distancing from the crafts, even though in many cases they initially learned it through contact with their parents or grandparents: “The children, for example, when they were little, during school holidays: ‘Dad, can I come with you?’ - ‘Sure.’ They would come, and indeed, when they were little, they would handle the clay, just like we did, just like we learned. But while we wanted to continue, they did not” (Imagery, 62 years old, male). Some participants mentioned that they do not see descendants in the family interested in continuing the craft practice, citing examples not only of their children but also of other relatives, such as siblings with children who likewise do not wish to continue working in the craft field. The perception of being alone in the activity also emerged in the accounts. In certain cases, artisans acknowledge that descendants enjoy the craft but do not want to pursue it. A lack of aptitude for the activity was also mentioned. Some artisans additionally compared their own experience with that of the younger generation, highlighting behavioral changes across generations.

The issue of “Low profitability and attractiveness of the activity” was also mentioned by the artisans (N3.1.2: n_cases_ = 14, n_references_ = 18), who notably highlighted that the craft practice requires a lot of work but provides little financial return: “As for what puts them off, I think part of it has to do with what you earn. It’s not something where you start now, for example, in this activity, and after one, two, 3 months you are already earning a salary. Trying to match it to a minimum wage is very difficult” (Imagery, 34 years old, male).

Moreover, the negative aspect of “Loss of continuity of the legacy” has emerged in the narratives of some participants (N3.1.3: n_cases_ = 10, n_references_ = 10), showing concern and sadness about the uncertainty of the future continuity of the legacy among descendants: “My daughters do not want to, they do not want to continue it. My brother’s daughter neither. So, look, when my brother and I are gone, the family’s art is finished” (Imagery, 62 years old, male).

The idea of “Conflict between respecting choices and the desire for continuity” also emerged among a number of participants (N3.1.4: n_cases_ = 8, n_references_ = 9), reflecting the ambivalence of the parents between respecting the descendants’ choices and wishing for the continuity of the craft practice: “I also do not have the courage to say, ‘Abandon your work and come to this,’ it would be a very big mistake on my part, but I think this should not end, I feel very sorry… I’ve always been sensitive to these arts, which I foresee will disappear, and I tried for the craft to stay in the family, so it would not die out, and I really encourage my daughter” (Weaving, 71 years old, male).

On the other hand, on the subcategory “Positive implications of lack of continuity” (N3.2), two subcategories have emerged: “Valuing the individual vocation” (N3.2.1: n_cases_ = 8, n_references_ = 10) refers to the acknowledgment that each descendant follows their own vocation or interest, reflecting respect for the individual path as well as the descendants’ preferences and motivation: “The real interest, the real passion. My youngest son is not interested in any of this, the oldest is a little interested, he still has a bit of interest; the youngest has none” (Basketry and Wicker, 45 years old, male). It also encompassed the perception that the continuity of the practice should not be forced: “In this art, either you come because you enjoy it, or if you come out of obligation, because you have to, if you are forced, I’ve said it before, in any art, a person only evolves if they like it. If they do not like it, they learn what they learned and stop there; they do not develop” (Wood, 62 years old, male). Other participants have expressed a “Sense of protection for descendants” (N3.2.2: n_cases_ = 5, n_references_ = 5) interpreting the lack of continuity of the activity as natural or even protective. Some statements show that certain parents consider that, when choosing a professional path, craft practice may be seen as demanding, poorly profitable, or with limited future prospects: “Everything was sold very cheaply, and it wasn’t enough to survive. No, it really wasn’t. Everyone chose other paths, and I also did not want to put my children into this, I said no, and well, it was just me left” (Imagery, 74 years old, female).

Despite this, it was highlighted that, in previous years, recognition of the practice was lower, which made the activity more challenging.

## 4 Discussion

This study explores how the family transmission of crafts and folk art may be linked to family relationships and cohesion in a UNESCO Creative City, both in cases of continuity of the activity across generations and when descendants did not pursue it professionally. The study combined quantitative and qualitative data, including walking interviews in the ateliers, field notes, and the application of the Family APGAR scale. This approach allowed methodological triangulation, reinforcing the robustness and scientific rigor of the results. In this way, the study adds new dimensions to the understanding of crafts and folk art and contributes to the existing literature on the subject.

### 4.1 Implications of family transmission of craft practice on family relationships and cohesion: positive vs. negative

Among the positive implications of family transmission of craft practice on family relationships and cohesion, an initial relevant aspect concerns the simultaneous presence of intergenerational and intragenerational dynamics within artisan families. Family collaboration and solidarity (N1.1) across different generations highlight the craft practice as a privileged space for the transmission of knowledge, sharing of experiences, and strengthening of emotional bonds among the artisans’ families. In parallel, intragenerational collaboration and solidarity, among siblings or spouses, also is perceived as contributing to family cohesion. Likewise, discourses of valuing, pride, and family recognition of craft work (N1.4) emerge in both intergenerational and intragenerational relationships, underscoring the affective dimension of the practice among these families.

This appreciation is reflected in positive intergenerational relationships and support from descendants, and is associated with better family functioning and reinforcing family relationships and cohesion. This pattern is also evident in statements referring to intergenerational family collaboration and solidarity in craft practice (N1.1.1), as well as in remarks expressing intergenerational valuing, pride, and recognition of craft work (N1.4.1), aspects that are associated with the continuity of the activity within the family. The results of the present study align with perspectives on intergenerational solidarity, suggesting that attachment to the family constitutes an important aspect of intergenerational family life, representing a lasting form of solidarity and a potential indicator of action and support (Bengtson and Roberts, 1991; Silverstein and Bengtson, 1997).

The family environment linked to craft practice as an enhancer of family relationships and cohesion (N1.3) suggests that family participation in the practice plays a central role in maintaining bonds and strengthening cohesion, functioning as a context for learning, mutual support, and appreciation of the cultural legacy. According to participants’ accounts, intergenerational relationships stand out as spaces of emotional, practical, and financial support, especially between parents and their descendants, contributing to family cohesion and well-being.

These results can be interpreted in light of intergenerational solidarity (Bengtson and Roberts, 1991) and Social Exchange Theory (Homans, 1958), as the domains of the scale reflect dimensions of affection, functional exchanges, and norms of reciprocity present in intergenerational relationships. Thus, joint involvement, shared decision-making, care, and commitment to others represent concrete forms of solidarity and exchange within the family, reinforcing both the continuity of craft practices and the cohesion of the family group. These findings resonate with the literature emphasizing the importance of family cohesion, which facilitates intergenerational transfers and enables the family to maintain and develop its legacy, strengthening family relationships and promoting cohesion (Fernández-Roca et al., 2014).

In global terms, there is growing recognition of the importance of intergenerational solidarity, grounded in the interdependence between generations throughout the life course and considered essential for building a society for all ages (United Nations Department of Economic and Social Affairs, 2025). The principle of intergenerational equity, which assigns responsibilities to present generations toward future ones, advocates balancing the short-term needs of current generations with the long-term needs of future ones, being intrinsically linked to the concept of sustainable development (Nações Unidas, 2018; United Nations Department of Economic and Social Affairs, 2023). This principle can be invoked to reflect on the continuity of the family legacy of craft and folk art, since the absence of succession in artisanal practice may be understood as depriving future generations of a cultural and identity dimension, emphasizing the broader relevance of preserving this family legacy across generations.

The results highlight the importance of the family craft legacy as a central element in maintaining the practice and reinforcing family relationships and cohesion. The construction and transmission of a legacy have been conceptualized as a relational experience of aging, associated with the desire to give meaning to life and to perpetuate one’s symbolic presence (Sousa et al., 2012), thereby strengthening bonds and family continuity. Consistently, other studies emphasize that leaving a legacy for younger generations constitutes a positive experience, generating joy and satisfaction for those involved and relating to expressions of generativity (Serrat et al., 2021).

Subcategory N1.2, which refers to the continuity of the family legacy, highlights the value of family transmission of the activity, the preservation of inherited know-how, learned techniques, and the memory associated with the practice, thereby strengthening bonds and intergenerational family relationships. In this context, it is evident that the importance of intangible cultural heritage does not lie solely in the cultural manifestation itself, but in the wealth of knowledge and skills it transmits from one generation to the next. Accordingly, intangible cultural heritage, as defined by UNESCO, is simultaneously traditional, contemporary, and living. In addition, this heritage is representative, community-based, and inclusive (UNESCO Intangible Cultural Heritage, 2003).

The results also indicate that affective memory and the multigenerational transmission of the craft legacy (N1.5) constitute a valued aspect of craft practice. Artisans’ statements reveal not only the recollection of moments shared with older generations but also the symbolic importance of objects, places, and expressions associated with the activity, functioning as markers of family identity. The significance of place in discussions of craft work is evidenced by the connection to origin, linking objects to the places and people who produced them (Bell and Vachhani, 2019). This dimension shows that craft practice transcends the technical and economic domains, involving the transmission of meanings and a relational and emotional character that strengthens intergenerational bonds.

These aspects are directly related to the domains assessed by the Family APGAR scale, which reflects five essential family functions: Adaptation, Partnership, Growth, Affection, and Resolve (Table 5).

**Table 5:** Family function domains identified in participants’ narratives through different thematic categories.

Family function domains*	Thematic categories
Adaptation	N1.6_Economic sustainability and family resilience through craft practice and the preservation of cultural heritage N2.1_Difficulties in continuity despite transmission N2.2_Low income and long working hours N2.3_Difficulties in balancing craft practice and family life N3.1_Negative implications of lack of continuity
Partnership	N1.1_Family collaboration and solidarity in craft practice N1.2_Continuity of the family legacy of craft and folk art in strengthening family relationships and cohesion N1.3_The family environment linked to craft as an enhancer of family relationships and cohesion N1.4_Valuing, pride, and family recognition of craft work N1.6_Economic sustainability and family resilience through craft practice and the preservation of cultural heritage
Growth	N1.1_Family collaboration and solidarity in craft practice N1.2_Continuity of the family legacy of craft and folk art in strengthening family relationships and cohesion N1.3_The family environment linked to craft as an enhancer of family relationships and cohesion N1.4_Valuing, pride, and family recognition of craft work N1.5_Affective memory and multigenerational transmission of the craft legacy N3.2_Positive implications of lack of continuity
Affection	N1.1_Family collaboration and solidarity in craft practice N1.2_Continuity of the family legacy of craft and folk art in strengthening family relationships and cohesion N1.3_The family environment linked to craft as an enhancer of family relationships and cohesion N1.4_Valuing, pride, and family recognition of craft work N1.5_Affective memory and multigenerational transmission of the craft legacy N3.2_Positive implications of lack of continuity
Resolve	N1.1_Family collaboration and solidarity in craft practice N1.6_Economic sustainability and family resilience through craft practice and the preservation of cultural heritage

*Domains identified with reference to the domains of the Family APGAR scale.

In this context, the literature on care work and gender inequalities may provide an interpretive perspective for understanding family dynamics. The literature highlights the need to recognize and value the care economy, emphasizing its role in the reproduction of everyday life and its implications for gender inequalities, in line with the United Nations Sustainable Development Goals (Nações Unidas, 2018; Power, 2020). Previous research has shown that parents’ domestic behaviors influence children’s attitudes toward the gendered division of labor, with fathers’ involvement in household tasks representing an important factor in the development of more gender-egalitarian attitudes (Cano and Hofmeister, 2023).

Through the concept of the “second shift,” Hochschild and Machung (2012) highlighted inequalities in the distribution of domestic and care work within families, even in contexts where both members of the couple participate in the labor market. They further argue that experiences often interpreted as individual reflect broader economic and cultural transformations in the organization of work and gender roles (Hochschild and Machung, 2012).

Complementarily, the intersections between paid and unpaid work should be considered to achieve a more comprehensive understanding of gender inequalities, as unpaid work is frequently rendered invisible despite constituting an essential component of the economy. This includes care-related activities such as social reproduction, subsistence production, work in family businesses, and community support (Antonopoulos, 2009). Beyond the gender inequalities associated with domestic and care work traditionally performed by women, unpaid work constitutes a fundamental component of economic and social functioning. In this context, women are more frequently engaged in this type of work than men (Antonopoulos, 2009).

Recent research on unpaid care work suggests that its distribution remains unequal between men and women, disproportionately affecting women and mothers, although a partial shift away from the traditional division of childcare has been observed, with increasing paternal involvement in caregiving activities (Gencer et al., 2024).

In this context, these inequalities and caregiving dynamics help situate craft practice within family life as both a productive and relational activity, embedded in the social and material conditions of family work. These dynamics may also be reflected in relationships among family members.

The transmission of memories, affections, and meanings is perceived as contributing to a sense of belonging and cultural continuity within the family, aligning with Family APGAR results, particularly the Affection domain, which evaluates expressions of tenderness and emotional support among family members. Thus, craft practice can be understood not only as a productive activity but also as a space for affective expression, preservation of memories and meanings, reinforcing family relationships, cohesion, and cultural identity. This study aligns with and may contribute to analyses arguing that the affective traits of crafts emerge in specific moments and places, reflecting an affective and inclusive understanding of organizational craft practice (Bell and Vachhani, 2019).

In the context of the remaining subcategories, evidence is also observed, beyond the Affection domain, in other domains of the Family APGAR scale. The Partnership domain is reflected in the joint involvement and active sharing of decisions and responsibilities among family members. The Growth domain reflects the maturity and fulfillment achieved by family members through mutual support and guidance. The Resolve domain refers to the commitment to dedicate time and provide physical and emotional support to other members, including the sharing of resources and space. The narratives also reveal that artisans demonstrate a capacity for adaptation when facing challenges, reorganizing resources and coping with adversity collaboratively. Such capacity aligns with the Adaptation domain of the Family APGAR scale, which assesses how the family jointly deals with problems and changes. The quantitative results also indicate positive scores in this domain, reflecting an overall perception of a highly functional family, even in the face of difficulties.

Among both older and retired participants, Family APGAR scale results indicated higher levels of family functioning. The statements of older participants were expressive across several categories, both in intergenerational dimensions, for example, between parents and descendants or grandparents and grandchildren, and intragenerational dimensions, such as between spouses or siblings, reinforcing the perception of good family functioning associated with craft practice. In this regard, research indicates that, as individual’s age and their social networks shrink, family relationships become particularly relevant, with both positive and negative aspects of these relationships potentially influencing well-being (Thomas et al., 2017).

The qualitative results indicate that craft practice functions as an integrative element of family relationships, fostering family cohesion across affective, relational, and collaborative dimensions. The collaboration, solidarity, appreciation, and family pride observed in participants’ narratives reflect dynamics of sharing and mutual support that align with the domains of the Family APGAR scale, suggesting an affinity with aspects of healthy family functioning. They can be understood in light of Social Exchange Theory (Homans, 1958), which views social relationships as processes of reciprocity based on the exchange of material and symbolic resources, such as support, affection, prestige, or knowledge. In this context, family transmission of the activity can be understood as a form of exchange, in which older generations offer skills, values, and cultural identity, while younger generations reciprocate with recognition, continuity, or reinforcement of the symbolic presence of the older adults.

These results align with perspectives that emphasize the value of human engagement and work attitudes associated with craft in an increasingly technology-driven world, reflecting the importance of the relational and meaningful dimensions of artisanal work (Kroezen et al., 2021).

Within the economic dimension of the activity (N1.6), participants’ statements highlighted not only its importance as a source of income and family support, but also its association with enjoyment of craft practice and its relevance to the family. The narratives reveal contexts of joint effort, among siblings, spouses, or different generations, demonstrating the contribution of craft practice to family resilience and household sustenance. Craft activity is associated with both the economic sustainability of families and the preservation of cultural heritage, reinforcing intergenerational continuity and illustrating families’ efforts to build a sustainable future. This dimension is also connected to a generative aspect, expressed both in the care taken to maintain the activity for future generations and in the valuing and preservation of the legacy transmitted by parents as cultural heritage. In a previous study conducted with part of this sample, the sense of generativity among older artisans was already highlighted (Igreja et al., 2025a). The present study, which includes both older and younger participants, expands on this finding, showing that the sense of generativity manifests across multiple subcategories and appears to characterize the entire N1 category.

At the same time, the results indicate that activities experienced within the family context can function as a social determinant of health, promoting active and healthy aging through collaboration, solidarity, and knowledge sharing across generations. They also highlight the importance of a family environment that strengthens relationships and cohesion, and fosters appreciation, pride, and recognition of craftwork within the family. This interpretation is supported by the quantitative results, which reveal high levels of family functioning (Family APGAR 7–10) and the predominance of well-being as the main reported benefit (78.6%).

These findings support the potential of cultural prescription as a tool to value traditional knowledge, promote the continuity of craft skills, and enhance individual and family well-being.

In the context of the negative implications of family transmission of craft practice (N2), although less pronounced than the positive ones, they remain relevant for understanding the importance of family relationships and cohesion. Among continuity difficulties (N2.1), situations of reduced practice associated with the absence of a spouse and the aging of artisans stand out, highlighting significant vulnerabilities in maintaining the activity. In the territory, craft practice is primarily carried out by older adults, active beyond retirement and important vehicles for the family transmission of knowledge (Costa, 2024; Igreja et al., 2025b). It is also consistent with studies on arts and crafts that highlight the significant impact of economic, motivational, promotional, and support factors on the adoption of family craft businesses and on the valorization of local tradition and culture (Deb et al., 2022). In this context, considering the importance of fostering family craft activities while including these various factors, it becomes relevant to consider measures that value family participation and contribute to the preservation, sustainability, and continuity of craft practice, especially in light of aging, health issues, and the decrease of involved family members. On the other hand, low income and long working hours (N2.2) constitute another relevant dimension, evidenced in some statements highlighting the high workload and limited financial return, factors that may be linked to family time, family relationships and cohesion. Additionally, difficulties in balancing craft practice and family life (N2.3) were evident, especially when spouses do not work together or when family support is absent. These results underscore the importance of family incentives that promote fairer income and facilitate better time management between work and family life.

The literature on family dynamics and intergenerational transmission shows that gender roles play a relevant role in shaping family support structures and economic trajectories. In Portugal, studies on informal support networks highlight the importance of social factors in family support dynamics, emphasizing that such support is predominantly provided by women, thereby contributing to the reproduction of gender inequalities within families (Wall et al., 2001).

These factors may have influenced how the family transmission of crafts and folk art is experienced within families and, consequently, may help shape family relationships, family cohesion, and the meanings attributed to the continuity of these intergenerational practices.

Consistently, research on intergenerational transmission indicates that women’s participation in the labor market and attitudes toward gender roles can be transmitted across generations, influencing the labor-related decisions of younger generations (Farré and Vella, 2013; Stranges, 2022). A study on the intergenerational transmission of female labor market participation shows that this process occurs more frequently from mothers to daughters than from mothers to sons (Stranges, 2022). Moreover, maternal attitudes toward women’s roles in the family and labor market significantly shape children’s attitudes and may also influence their decisions and labor market participation (Farré and Vella, 2013).

Additionally, the intergenerational transmission of social norms suggests that women whose partners were raised by mothers with higher labor market participation are more likely to be employed, work longer hours, and earn higher incomes. However, this transmission does not necessarily lead to greater equality in the division of domestic labor, with a higher incidence of the double burden, in which women combine paid employment with domestic responsibilities (Schmitz and Spiess, 2022). Another study finds that parenting behaviors during childhood can have long-lasting effects on children’s behaviors, including in the context of paid employment. In particular, being raised by a mother who participates in the labor market is associated with women’s participation in the labor market in adulthood, suggesting that maternal employment behavior can shape daughters’ career trajectories over the life course (Van Putten et al., 2008).

Consistently, other studies that have used the Family APGAR scale, in Portugal and in other countries, show that highly functional families and perceived family support contribute to better quality of life, even in populations with different age characteristics or health conditions (Kaur et al., 2015; Prazeres and Santiago, 2016). Thus, craft practice, as a culturally significant activity carried out in the family context, can be understood as a promoter of active and healthy aging by fostering social engagement, valuing skills, transmitting knowledge, and strengthening relationships across generations. In this regard, its potential role in developing safeguarding strategies that address the challenges of aging, promote quality of life, and combat ageism stands out, in line with global guidelines for active and healthy aging (World Health Organization, 2002; United Nations, 2020; World Health Organization, 2020, 2021; United Nations, 2023). Ageism weakens intergenerational solidarity by using age to categorize and divide people in harmful ways (World Health Organization, 2021), which reinforces the importance of valuing the experience of older adults, fostering social participation, and strengthening intergenerational bonds.

### 4.2 Implications of the lack of continuity of craft practice for family relationships and cohesion: positive vs. negative

The implications of the lack of continuity of craft practice, both negative and positive, emerge in family relationships and in the preservation of the legacy, being mentioned by more than half of the participants. Negative implications (N3.1) were particularly prominent in the artisans’ narratives. The disinterest or disengagement of descendants (N3.1.1) show that discontinuity is experienced ambivalently. There is a desire to see the legacy continue, accompanied by recognition and concern regarding the lack of interest among descendants. This perception reflects not only concern about the future of the practice but also, for several artisans, an emotional experience marked by the feeling of being alone in the practice.

Previous studies indicate that family support constitutes a powerful source of motivation, capable of enhancing work performance, especially when intrinsic motivation is low (Menges et al., 2016). In line with the literature on creativity and organizational innovation, which highlights the interaction among intrinsic motivation, environmental factors, and individual skills as the basis of the creative process (Amabile, 1988), family support can be understood as one of these contextual factors that strengthen descendants’ engagement and foster the continuity of craft activity. Thus, family motivation can reinforce involvement in the activity even when intrinsic interest is low, enhancing acquired skills, since, despite descendants’ disengagement (N3.1.1), the narratives show several instances of skill acquisition through collaboration in the family practice. Furthermore, dimensions such as collaboration, mutual support, and recognition in craft work were shown to contribute to family cohesion and satisfaction.

The theme of low profitability and attractiveness (N3.1.2) emerged as one of the main reasons associated with the lack of continuity, being reported by both younger and older artisans, who perceive the risk of losing the continuity of the family legacy. Thus, while familial and relational factors constitute important elements for descendants’ motivation and engagement, structural and economic aspects also prove to be decisive, emerging not only as a negative implication in family transmission but also as one of the causes for the lack of continuity of the practice. This impact is particularly evident in the motivation of new generations to engage in the practice, with repercussions for family relationships and cohesion. This finding highlights the relevance of measures that could enhance the attractiveness of craft and folk art practices, through the valorization and fair remuneration of products, as well as the implementation of strategies that strengthen the sector’s competitiveness, differentiating it through its authenticity. The literature on these practices in the study territory indicates that artisans are recognized as having the right to better living conditions, providing fairer compensation for their work, and that their cultural production is considered heritage to be preserved and valued both culturally and economically for the future (Município de Barcelos, 2015). Many artisanal traditions have long-standing teaching and learning systems, which can be strengthened through financial incentives for learners and instructors, making knowledge transmission more attractive for both parties (UNESCO, 2025).

Furthermore, these findings align with previous studies on family crafts, which identified suitable promotional tools, such as appropriate pricing, maintaining product quality, and identifying customer needs, as vital factors for the adaptation of family craft businesses (Deb et al., 2022). In this way, they also appear to respond to another theme highlighted in the statements, namely concerns about the potential loss of continuity of the legacy (N3.1.3).

The study includes the participation of both older and younger members of the community, professionally engaged in crafts and folk art, and may contribute to the Sustainable Development Goals, as exemplified by SDG 8, Decent Work and Economic Growth, which promotes policies aimed at development, support for productive activities, creativity and innovation, as well as the valorization of culture and local products. In addition, it aligns with measures for active and healthy aging and with the Decade of Healthy Aging 2020–2030 (World Health Organization, 2002; Nações Unidas, 2018; United Nations, 2020; World Health Organization, 2020, 2021; United Nations, 2023). Measures such as including information on the pieces, for example, the number of hours the artisan dedicated to creating the product, details on family and intergenerational collaboration, as well as strategies that recognize and value these contributions, such as intergenerational learning incentive programs, initiatives to enhance artisanal skills, or support for the formalization of family businesses, can enrich the perceived value of creations, strengthen family dynamics, and serve as a stimulus for other individuals and families. Such measures can contribute to the continuity, sustainability, and greater recognition of craft activity, while also advancing the discussion in the literature on this topic.

The conflict between respecting descendants’ choices and the desire for continuity (N3.1.4) reflects parental ambivalence, as they balance respect for their descendants’ decisions with the wish to maintain the family legacy. This tension highlights challenges in the context of intergenerational transmission. This theme was clearly evidenced with the emergence of the subcategory positive implications of lack of continuity (N3.2), encompassing themes related to valuing individual vocation and the sense of protection for descendants. In the context of the present study, valuing individual vocation (N3.2.1) respecting the vocation and personal choices of descendants, can help mitigate potential generational tensions. Previous studies based on the concept of ambivalence suggest that tensions arise and persist within families regardless of their pattern of intergenerational relationships, and can manifest in terms of interests, feelings, values, and interpersonal relationships. Even in traditionally supportive families, each generation seeks to follow its own path, and family strategies may function to alleviate these tensions (Brannen, 2006).

This appreciation of individual paths reflects a family dynamic characterized by acceptance and recognition of autonomy, which also constitutes a positive aspect for family relationships and cohesion, aligning with the data obtained from the Family APGAR scale, in which families were classified as highly functional. Furthermore, a positive correlation was observed between age and family functioning, suggesting that older participants tend to report more favorable perceptions, which may reflect the importance of supportive and accepting family dynamics for positive functioning.

In the context of the sense of protection for descendants (N3.2.2), the statements revealed that some parents discourage engagement in the practice, often perceiving it as low-paying and seeking to protect their descendants from the difficulties associated with the activity. At the same time, they express satisfaction with their children’s educational and professional paths, recognizing their achievements. Consistent with intergenerational solidarity (Bengtson and Roberts, 1991) and Social Exchange Theory (Homans, 1958), these behaviors reflect affective, functional, and normative dimensions in family relationships, exemplifying the exchange of symbolic resources and care across generations. In this context, the generative intent of these parents is also evident, as they seek to protect their descendants while valuing the family legacy and supporting the development of future generations. This attitude corresponds to domains of the Family APGAR scale, such as Growth, related to physical, psychological, and emotional maturity as well as the achievements attained by family members through mutual support and guidance, and Affection, which refers to the existence of caring and nurturing relationships among family members.

It is important to highlight that some participants emphasized that, in recent years, there have been improvements, particularly associated with greater recognition of the practice. This recognition may be related to the benefits arising from Barcelos being designated a UNESCO Creative City in the category of crafts and folk art, as well as to the valorization efforts promoted at the municipal level (UNESCO, 2017; Município de Barcelos, 2023). Such advancements constitute an important indicator, as they can motivate descendants with an interest in the field as well as others drawn to it, contributing to the preservation of practices in crafts and folk art. In line with the results discussed, these changes underscore the importance of supporting artisans and families, encouraging younger generations to consider the field as a professional option or complementary activity.

In this context, traditional handicrafts have significant historical and cultural importance, constituting an essential element of the intangible cultural heritage of communities and ethnic groups around the world and playing a central role in the sustainable transmission of this heritage at a global level (Ding et al., 2025).

Despite the study’s contributions, its limitations must be acknowledged: the sample was limited to a single city, and the qualitative analysis depended on participants’ expressiveness. Additionally, the study did not collect information on who assumes primary financial responsibility within the household, nor on the distribution of gender roles within it, including family responsibilities, which may limit the analysis of possible gender dynamics associated with the intergenerational transmission of craft practices. Similarly, it does not address the existence and distribution of caregiving responsibilities within the family, including care for children, older adults, and dependents, which limits understanding of the conditions of availability, participation, and transmission of craft practices. Information regarding participants’ ethnic or racial background was also not collected. Although the group in the present study is likely to be relatively homogeneous, future studies may benefit from including and analyzing this dimension, taking into account dynamics of mobility and migration.

As Crenshaw (1989) argues, the isolated analysis of social categories such as gender and race may render experiences situated at their intersection invisible, thereby constraining the understanding of socially structured inequalities (Crenshaw, 1989). Furthermore, literature on race, ethnicity, and culture in caregiving research shows that care experiences and outcomes may vary across racial and ethnic groups, highlighting the importance of considering factors such as values, beliefs, cultural norms, and processes of acculturation and assimilation (Dilworth-Anderson et al., 2002).

In this sense, research on intangible cultural heritage suggests that the transmission and safeguarding of traditional handicrafts occur in diverse cultural and socioeconomic contexts, which may influence the forms of continuity and valorization of these practices (Ding et al., 2025), indicating that these dimensions may shape how intergenerational transmission is experienced.

This aspect is particularly relevant in light of UNESCO principles related to inclusion, well-being, and the promotion of cultural diversity, as access, participation, and valuation of intangible cultural heritage may vary across different social and cultural groups (UNESCO, 2023; UNESCO Intangible Cultural Heritage, 2003). Future studies should include more diverse samples, compare regional and cultural contexts, incorporate a more in-depth analysis of gender dynamics within families, including the distribution of economic responsibility, and consider longitudinal approaches to deepen understanding of how family legacy evolves and how it influences family relationships and cohesion, as well as how intergenerational solidarity contributes to the preservation of intangible cultural heritage.

### 4.3 Conclusion

This study, conducted with a sample of professional artisans from a UNESCO Creative City of crafts and folk art in Portugal, explored how family transmission of artisanal practices relates to family cohesion and relationships. The findings offer insights into this transmission and its implications for cohesion, family relationships, generativity, and the development of actions that promote the continuity and valorization of crafts, expanding previous knowledge on the topic. The analysis identified multiple perceived implications, with predominantly positive dynamics such as collaboration, intergenerational solidarity, and the continuity of the legacy. In contrast, the lack of continuity in the practice was associated with negative experiences, including the disengagement of descendants and the loss of legacy continuity.

The study highlights that the family transmission of crafts and folk art, beyond serving as a key resource for safeguarding intangible cultural heritage and valuing artisans, may be associated with family cohesion and intergenerational well-being. In a context of global aging, fostering sustainable practices that strengthen family dynamics, supported by cultural valorization and economic sustainability initiatives, becomes essential for stimulating the interest of younger generations. Crafts and folk art, beyond their professional dimension, function as family-transmitted cultural practices that may contribute positively to social determinants of health and to well-being.

## Data Availability

The raw data supporting the conclusions of this article will be made available by the authors, without undue reservation.
