# Supplementary material for: Family transmission of crafts and folk art: a mixed-methods study on family relationships and cohesion among artisans in a UNESCO Creative City
Source: Front Sociol. 2026 Jun 17;11:1800473. doi: 10.3389/fsoc.2026.1800473 (PMC13318937; doi:10.3389/fsoc.2026.1800473)
Supplement: Supplementary file 1 [file Data_Sheet_1.PDF]

Topics addressed in the walking interview guide

The list below presents the topics addressed in the walking interview guide, as explored through participants' spontaneous discourse.

Interview guide topics
(a) the participants' path in craft and folk art<br>(b) history of the activity carried out; pieces created; the atelier; and whether the atelier is located next to the living space<br>(c) feelings of belonging to the territory<br>(d) what most characterizes their artistic work<br>(e) days of the week worked; average number of pieces created per day; and average time required per piece<br>(f) different types of pieces that have been created<br>(g) whether the person has always been engaged in the activity or whether there has been a change over time<br>(h) materials and raw materials used in the craft activity<br>(i) existence of specific expressions commonly used in the activity<br>(j) how the workweek unfolds; what characterizes a good workday and a less positive one<br>(k) more demanding and less demanding types of work<br>(l) whether the activity comes from previous generations and if there are other family members professionally active in the sector<br>(m) whether the start of their craft activity was spontaneous or influenced by someone else<br>(n) participation of younger generations in the activity<br>(o) to what extent they had the opportunity to help prepare younger generations, family members, or others for the sector's activities<br>(p) what it means to have had or not had this opportunity<br>(q) to what extent the artistic activity has influenced family relationships<br>(r) what causes younger generations to approach or distance themselves from the activity in the sector<br>(s) to what extent they feel good about life and consider themselves to have quality of life<br>(t) what allows or would allow the person to feel good about life<br>(u) whether the person considers themselves to be healthy<br>(v) how satisfied they are with their health<br>(w) health situations that may have affected their life or work<br>(x) how the artistic activity affects (positively and/or negatively) their quality of life and well-being<br>(y) whether the activity contributes to personal satisfaction/self-esteem<br>(z) what motivates them to continue their work in the sector<br>(aa) what they value most in the work they do<br>(bb) whether they plan to stop working or making pieces<br>(cc) whether they consider what they do to be relevant<br>(dd) whether they have noticed changes over the years regarding the creative meaning of their activity<br>(ee) whether there have been changes over the past few years in how they perform their work or how they view crafts and folk art<br>(ff) whether they actively participate in local, national, or international dynamics, and if they participate in events related to craft and folk art<br>(gg) whether they engage in other social participation activities<br>(hh) what the artistic activity they perform means to them
