# Supplementary material for: Family transmission of crafts and folk art: a mixed-methods study on family relationships and cohesion among artisans in a UNESCO Creative City
Source: Front Sociol. 2026 Jun 17;11:1800473. doi: 10.3389/fsoc.2026.1800473 (PMC13318937; doi:10.3389/fsoc.2026.1800473)
Supplement: Supplementary file 2 [file Data_Sheet_2.pdf]

Consolidated criteria for reporting qualitative studies (COREQ): 32-item checklist

Topic and item no.	Guide questions/description	Reported on page no.
**Domain 1: Research team and reflexivity**		
**Personal Characteristics**		
**1. Interviewer/facilitator**	**Which author/s conducted the interview or focus group?**	The interview was conducted by Author 1 (Sandra Igreja).
**2. Credentials**	**What were the researcher's credentials? E.g. PhD, MD3.**	**Sandra Igreja**<br>PhD Candidate in Gerontology and Geriatrics; MEd, Educational Sciences (specialization in Education and Artistic Expression).<br>**Constança Paúl**<br>Aggregation in Psychology; PhD Biomedical Sciences, expertise in Psychology; Master in Biomedical Sciences; Degree in Psychology.<br>**Soraia Teles**<br>PhD Clinical and Health Services Research;<br>MEcon, Social Economics;<br>MSSc, Health and Clinical Psychology.
**3. Occupation**	**What was their occupation at the time of the study?**	PhD Candidate and municipal officer in social and health services (Department of Education, Health, and Social Services) (SI);<br>Researcher and Full Professor (CP);

		Researcher (ST).
**4. Gender**	**Was the researcher male or female?**	Female
**5. Experience and training**	**What experience or training did the researcher have?**	Experience and training in both qualitative and quantitative data collection and analysis.
**Relationship with participants**		
**6. Relationship established**	**Was a relationship established prior to study commencement?**	No prior relationship with participants.
**7. Participant knowledge of the interviewer**	**What did the participants know about the researcher? e.g. personal goals, reasons for doing the research.**	Participants were provided with a comprehensive information sheet that included the study's aim, participation details, the responsible researchers and contact information (see Section 2.2: Participants and recruitment).
**8. Interviewer characteristics**	**What characteristics were reported about the interviewer/facilitator? e.g. Bias, assumptions, reasons and interests in the research topic**	Participants were informed about both the interviewer and the research team (via a comprehensive information sheet), including their names and professional affiliations. The interviewer's interest in the topic stemmed from her academic background and professional experience.
**Domain 2: study design**		
**Theoretical framework**		

**9. Methodological orientation and theory**	**What methodological orientation was stated to underpin the study? e.g. grounded theory, discourse analysis, ethnography, phenomenology, content analysis**	The study followed a mixed-methods design with methodological triangulation, combining quantitative analysis of survey data with qualitative content analysis of walking interview narratives (see Section 2: Material and methods; 2.1 Study design, 2.4 Data analysis, 2.4.1 Statistical analysis, 2.4.2 Content analysis).
**Participant selection**		
**10. Sampling**	**How were participants selected? e.g. purposive, convenience, consecutive, snowball**	Purposive, non-probabilistic sampling was used to recruit professional artisans active in ateliers located in Barcelos, Portugal, who met the inclusion criteria described in Section 2.2 (see Section 2.2: Participants and recruitment).
**11. Method of approach**	**How were participants approached? e.g. face-to-face, telephone, mail, email**	Participants were initially approached by telephone to provide information about the study, assess preliminary interest, and evaluate eligibility. Those who agreed to participate were subsequently scheduled for face-to-face data collection in their ateliers (see Section 2: Materials and methods – 2.2 Participants and recruitment).

**12. Sample size**	**How many participants were in the study?**	Seventy professional artisans (*n* = 70) participated in the study (see Section 3: Results – 3.1 Statistical analysis (quantitative data); 3.1.1 Sociodemographic and professional characteristics).
**13. Non-participation**	**How many people refused to participate or dropped out? Reasons?**	Of the artisans who responded to the initial contact, 21 did not participate. Reasons for non-participation included no longer being active in their craft, illness, or not meeting the eligibility criteria.<br>Among those who agreed to take part in the study (*n* = 72), data collection was completed once all eligible participants had been interviewed. None dropped out during the study. Two participants were later excluded from the final analysis for not reporting family transmission of the craft activity, a specific inclusion criterion of the present substudy, integrated within a broader research project. (see Section 2: Materials and methods – 2.1 Study design; 2.2 Participants and recruitment; Section 3: Results – 3.1 Statistical

		analysis (quantitative data); 3.1.1 Sociodemographic and professional characteristics).
**Setting**		
**14. Setting of data collection**	**Where was the data collected? e.g. home, clinic, workplace**	Data collection was conducted in the participants' ateliers.<br>(see Section 2: Materials and methods – 2.2 Participants and recruitment).
**15. Presence of non-participants**	**Was anyone else present besides the participants and researchers?**	In general, no individuals other than the participants and the researcher were present during data collection. However, during the walking interviews, the presence of other individuals was observed in some cases, particularly among participants from the Imagery, Iron and Derivatives, and Pottery sectors. These individuals were located in different areas of the ateliers, engaged in painting or craft-related activities (e.g., Barcelos Rooster figures, pottery pieces). Most were family members involved in the artisanal production process. Their presence was expected and contextually relevant, as

		family involvement in the craft activity constituted part of the interview focus and later emerged as a theme in the results. (see Section 2: Materials and methods – 2.3 Instruments; Section 3: Results – 3.1.2 Family characteristics and functioning; 3.2.1 Positive and negative implications of family transmission of craft practice on family relationships and cohesion; 3.2.2 Positive and negative implications of lack of continuity of craft practice on family relationships and cohesion).
**16. Description of sample**	**What are the important characteristics of the sample? e.g. demographic data, date**	The study included 70 professional artisans. Sociodemographic characteristics (Table 1) comprised age, gender, marital status, years of education, professional training, retirement status, main professional activity, primary source of income, monthly income. Professional characteristics (Table 2) included the craft sector, age at the start of the craft activity, years of

		residence in the parish, atelier location at the residence, weekly hours dedicated to the activity, hours spent on non-professional activities, and the main perceived benefits of the activity (economic, health, quality of life, well-being, social status).<br>Family characteristics of participants and members involved in craft and folk art activities, and perception of family functioning (Family APGAR scale) (Table 3) covered household size; offspring, number of offspring, grandchildren, and great-grandchildren; household composition; family members involved in craft activities; regular engagement in craft activities; and regular engagement in craft activities by kinship relationship. Perceptions of family functioning were assessed using the Portuguese-adapted Family APGAR scale.<br>Questions related to family transmission included whether the activity had been transmitted from previous

		generations and whether the artisan is currently transmitting the craft activity to younger generations.<br>Data collection took place in the artisans' ateliers during the first quarter of 2024.<br>(see Section 2: Materials and methods – 2.2 Participants and recruitment; Section 3: Results – 3.1 Statistical analysis (quantitative data), 3.1.1 Sociodemographic and professional characteristics, 3.1.2 Family characteristics and functioning.
**Data collection**		
**17. Interview guide**	**Were questions, prompts, guides provided by the authors? Was it pilot tested?**	Yes. The interview guide was developed by the authors specifically for this study; it was not pilot tested.<br>The present study aimed to analyze how the family transmission of artisanal practices is associated with family relationships and cohesion, based on a sample of artisans from Barcelos (Portugal), a UNESCO Creative City of Crafts and Folk Art.<br>The interview guide included main and secondary questions. Data collection

		employed the Go-along Walking Interview method to capture contextualized narratives and observations within participants' work environments.<br>(see Section 2: Materials and methods – 2.3 Instruments).
**18. Repeat interviews**	**Were repeat interviews carried out? If yes, how many?**	Yes, two repeat interviews were carried out to clarify information.
**19. Audio/visual recording**	**Did the research use audio or visual recording to collect the data?**	Yes, the interviews were audio-recorded after obtaining participants' consent (see Section 2 Materials and methods – 2.2. Participants and recruitment, 2.3 Instruments).
**20. Field notes**	**Were field notes made during and/or after the interview or focus group?**	Yes, field notes were taken during the walking interviews whenever deemed essential to provide better contextualization and understanding of the information collected. This approach aligns with the study's focus on capturing contextual and non-verbal cues to enrich the analysis (see Section 2 Materials and methods – 2.3 Instruments).
**21. Duration**	**What was the duration of the interviews or focus group?**	The interviews lasted between 1 and 2 hours (see Section 2

		Materials and methods – 2.3 Instruments).
**22. Data saturation**	**Was data saturation discussed?**	Yes, data saturation was discussed among the researchers. Saturation was considered to have been reached when no new themes emerged, and the information became redundant.
**23. Transcripts returned**	**Were transcripts returned to participants for comment and/or correction?**	No, transcripts were not returned to participants for comment or correction.
**Domain 3: analysis and findings**		
**Data analysis**		
**24. Number of data coders**	**How many data coders coded the data?**	Two researchers (Researcher A and Researcher B) independently coded the textual data sources (see Section 2 Materials and methods – 2.4 Data analysis, 2.4.2 Content analysis).
**25. Description of the coding tree**	**Did authors provide a description of the coding tree?**	Yes. Table 4 (see Section 2 Materials and methods – 2.4 Data analysis, 2.4.2 Content analysis; 3 Results – 3.2 Content analysis (qualitative data)).
**26. Derivation of themes**	**Were themes identified in advance or derived from the data?**	The themes were derived from the data in an inductive manner.<br>(see Section 2 Materials and methods – 2.1 Study design, 2.4 Data analysis, 2.4.2

		Content analysis; Section 3 Results – 3.2 Content analysis (qualitative data), 3.2.1 Positive and negative implications of family transmission of craft practice on family relationships and cohesion; 3.2.2 Positive and negative implications of lack of continuity of craft practice on family relationships and cohesion).
**27. Software**	**What software, if applicable, was used to manage the data?**	NVivo was used to support content analysis, while SPSS was employed to manage quantitative data and generate descriptive statistics.<br>(see Section 2 Materials and methods – 2.4 Data analysis, 2.4.1 Statistical analysis, 2.4.2 Content analysis).
**28. Participant checking**	**Did participants provide feedback on the findings?**	No. (see Section 2 Materials and methods – 2.2 Participants and recruitment, 2.3 Instruments).
**Reporting**		
**29. Quotations presented**	**Were participant quotations presented to illustrate the themes/findings? Was each quotation identified?**<br>**e.g. participant number30**	Yes, for both questions. Participant quotations were presented to illustrate the themes and findings, each identified with relevant information (e.g., craft sector, age, gender), such as (Imagery, 84 years old, male).

		See Table 4 and the 3 Results section (3.2 Content analysis (qualitative data), 3.2.1 Positive and negative implications of family transmission of craft practice on family relationships and cohesion; 3.2.2 Positive and negative implications of lack of continuity of craft practice on family relationships and cohesion).
**30. Data and findings consistent**	**Was there consistency between the data presented and the findings?**	Yes. 3 Results section. There was consistency between the data presented and the findings. Quantitative results were clearly reported and interpreted in line with the study's objective (3 Results, 3.1 Statistical analysis (quantitative data), 3.1.1 Sociodemographic and professional characteristics, Table 1 and Table 2; 3.1.2 Family characteristics and functioning, Table 3. Qualitative findings were supported by illustrative quotations that aligned with the thematic categories identified (Table 4); 3 Results, 3.2 Content analysis (qualitative data), 3.2.1 Positive and negative

		implications of family transmission of craft practice on family relationships and cohesion; 3.2.2 Positive and negative implications of lack of continuity of craft practice on family relationships and cohesion.
**31. Clarity of major themes**	**Were major themes clearly presented in the findings?**	Yes, both quantitative and qualitative findings were clearly presented, with major themes illustrated through participant quotations and supported by narrative explanations. These themes were also interpreted in the discussion section (3 Results, 3.1 Statistical analysis (quantitative data), 3.1.2 Family characteristics and functioning; 3.2 Content analysis (qualitative data), 3.2.1 Positive and negative implications of family transmission of craft practice on family relationships and cohesion; 3.2.2 Positive and negative implications of lack of continuity of craft practice on family relationships and cohesion; 4 Discussion, 4.1 Implications of family transmission of craft practice on family relationships and

		cohesion: positive vs. negative; 4.2 Implications of the lack of continuity of craft practice for family relationships and cohesion: positive vs. negative; 4.3 Conclusion).
**32. Clarity of minor themes**	**Is there a description of diverse cases or discussion of minor themes?**	Yes. Minor themes and less frequent perspectives were presented in the results and discussed in the interpretation. Each category and subcategory emerging from the content analysis was discussed regardless of the frequency or predominance of the responses. This approach allowed the inclusion of less expressive viewpoints, enriching the understanding of the phenomenon (3 Results, 3.2 Content analysis (qualitative data), 3.2.1 Positive and negative implications of family transmission of craft practice on family relationships and cohesion; 3.2.2 Positive and negative implications of lack of continuity of craft practice on family relationships and cohesion; 4 Discussion, 4.1 Implications of family transmission of craft practice

		on family relationships and cohesion: positive vs. negative; 4.2 Implications of the lack of continuity of craft practice for family relationships and cohesion: positive vs. negative).
